# An institutional audit of the use of novel drugs in pediatric oncology

**DOI:** 10.1002/cnr2.1404

**Published:** 2021-05-03

**Authors:** Justin Lee, Lynn Gillam, Sarah Kouw, Maria C. McCarthy, Jordan R. Hansford

**Affiliations:** ^1^ Children's Cancer Centre, Royal Children's Hospital Parkville Victoria Australia; ^2^ Neurodisability and Rehabilitation ‐ Clinical Sciences, Murdoch Children's Research Institute Parkville Victoria Australia; ^3^ Department of Pediatrics University of Melbourne Melbourne Victoria Australia; ^4^ Department of Human Bioethics University of Melbourne Melbourne Victoria Australia; ^5^ Brain and Mind ‐ Clinical Sciences Murdoch Children's Research Institute Parkville Victoria Australia; ^6^ Cancer ‐ Cell Biology Murdoch Children's Research Institute Parkville Victoria Australia

**Keywords:** clinical cancer research, experimental therapeutics, medical oncology, pediatric cancer

## Abstract

**Background:**

Significant challenges persist in treating children with rare, relapsed, or refractory malignancies. Novel molecularly targeted drugs promise improved outcomes for these children with reduced toxicity. However, there is often limited evidence to substantiate their clinical efficacy and guide their use. This raises issues for clinical decision‐making, ethical concerns surrounding equity of access to these often‐expensive agents, and the management of families' expectations for cure. This audit evaluated the off‐label use of novel drugs and associated clinical outcomes in order to guide the development of future clinical and ethical guidelines.

**Aim:**

To evaluate the patterns in the off‐label use of novel drugs for treating childhood cancer and the associated clinical outcomes to guide prospective studies and inform ethical and clinical governance protocols for the use of these agents.

**Methods:**

A retrospective audit was performed for all patients who received novel drugs off‐label as treatment for their malignancy at an Australian pediatric oncology center between 2010 and 2019.

**Results:**

One hundred patients with 32 unique diagnoses received 133 novel drugs across 124 regimens. Eighty‐four patients received these drugs at the second line of treatment or greater. Novel drug median cost was $15 521 AUD (Range: $6.53 AUD to $258 339 AUD) and was primarily funded by the hospital (*N* = 60/133, 45.1%) or compassionate access from pharmaceutical companies (*N* = 52/133, 39.1%). Decision‐making related to novel drugs was inconsistently documented. Ninety‐one of 124 treatment regimens commenced between 2010 and 2019 resulted in objective responses (73.4%), but only 35 were still ongoing upon review in June 2020 (38.5%). Median response duration was 12.6 months (Range: 0‐93.2 months).

**Conclusions:**

While novel drugs were largely unable to definitively cure patients, most achieved objective responses. Prospective trials and more rigorous documentation are needed to fully inform the future use of these agents given the heterogeneity of their applications.

## 1 INTRODUCTION

Significant challenges persist in improving treatment outcomes for children with rare, relapsed, or refractory malignancies.^1^ Intensifying traditional chemotherapeutic regimens achieves little benefit at the cost of significant toxicity,^1^ highlighting the need for new treatment approaches. Over the past decade, technological advancements have greatly facilitated the expansion of precision medicine, a novel treatment paradigm integrating advanced molecular analytical techniques into the diagnosis and personalized treatment of malignancies.^2^ By targeting cancer‐specific biomarkers and genomic changes, precision medicine promises greater clinical benefit without increase in toxicity.

Despite this theoretical potential, there is currently limited evidence to substantiate the efficacy of novel drugs and guide their optimal use in clinical practice.^3^ Few clinical trials have been undertaken to evaluate these agents. Owing to the rarity of pediatric cancers and few eligible patients,^3^ clinicians have often relied on off‐label or compassionate‐use programs to access these drugs.^4^, ^5^ However, the significant costs of these novel agents have raised ethical issues surrounding their widespread implementation and accessibility to all patients.^6^ The limited evidence for the clinical efficacy also makes it challenging to justify funding drugs through government mechanisms,^6^ and to manage families' expectations for cure.^7^

The off‐label use of novel drugs is undertaken in our pediatric oncology center, particularly for relapsed and refractory diseases, despite a paucity of data around their use. In this context, it remains unclear whether these novel drugs provide enough benefit that their off‐label use should be routine in pediatric oncology or if they are best restricted to a controlled clinical trial setting. The aim of this study therefore was to evaluate current practices of prescribing novel drugs off‐label to pediatric patients at a large tertiary pediatric oncology center, including the associated clinical outcomes, in order to inform future studies and provide clinical and ethical recommendations for the use of these medications.

## 2 METHODS

### 2.1 Study design and research ethics

We conducted a retrospective audit at a large tertiary pediatric hospital utilizing patient data extracted from the hospital electronic medical record (EMR). Ethics approval was gained from the RCH Human Research Ethics Committee (HREC) to access the required data from the EMR records (QA/60416/RCHM‐2019). For retrospective audits, it is not a requirement of the HREC to obtain informed consent from patients or guardians where the audit involves the use of existing de‐identified clinical data with no foreseeable risk to the participants. In this study, the HREC deemed this to be the case.

### 2.2 Study population

All patients who received a novel drug off‐label for the treatment of their malignancy between 2010 and 2019 were included in the study. Novel drugs were defined as medications, which were considered above the “standard‐of‐care” for their prescribed indication by a senior clinical oncologist. Patients who accessed novel drugs through clinical trials were not considered eligible for this study, given the stringent governance and reporting procedures in the clinical trial setting.

### 2.3 Data collection

In the Australian healthcare system, drugs are traditionally dispensed by the hospital at little to no cost to the patients through the Pharmaceutical Benefits Scheme (PBS). For drugs for which approved governmental funding is not available, the hospital Drug Usage Committee (DUC) determines patient eligibility for hospital‐funded access to medication on a case‐by‐case basis. As such, eligible patients were identified using the pharmacy dispensary database and the EMR. Data, including patient and parents' demographics, were extracted from the clinical notes stored on the EMR. Each patient was assigned a unique study ID and data were stored using REDCap electronic data capture tools on a secure server.^8^

Disease data collected included diagnosis, tumor classification, and date of treatment allocation as well as disease state (relapsed, refractory or other) and line of treatment at the time of novel drug prescription. If a novel drug was added to an existing treatment regimen, this was considered another line of treatment. Data extracted to analyze the patterns of prescription for these novel drugs included date of prescription, drug name, billing type, and the overall cost of the medication in Australian dollars (AUD). Outcome data were recorded, including response type, defined according to the documented interpretation of radiographic results including any criteria cited to describe clinical response. Clinical response descriptions were used only where no imaging results were available. Response durations were defined as the time between the first documentation of response to the first documentation of disease progression, recurrence, and patient death or, in the case of ongoing responses, at the time of review, June 2020.

To further characterize the clinical practice of prescribing novel drugs, additional details were audited for example, prescribing consultant, whether there was external consultation and/or multidisciplinary meeting discussion. The DUC meeting minutes from 2010 to 2019 were also systematically reviewed to assess factors, which influenced drug allocation as well as the quality of documentation surrounding each decision. Following data collection, deidentified patient data were exported from the REDCap database for descriptive statistical analysis.

## 3 RESULTS

### 3.1 Patient demographics

One‐hundred and twenty‐one patients were identified for study inclusion. Twenty‐one patients were excluded owing to insufficient documentation prior to the introduction of an EMR in 2016. The remaining 100 patients were prescribed a total of 133 drugs across 124 treating regimens, where nine treatment regimens combined two novel drugs. Twenty‐eight patients received more than one novel drug in total: 23 were prescribed two, and five were prescribed three. Patient demographic details are summarized in Table 1.

**TABLE 1:** Summary of patient demographics and disease details

Patient demographics (*N* = 100)	*N* (%)
**Sex**	
Male	52 (52.0)
Female	48 (48.0)
**Age (years)**	
Median	6.71
Range	0.010‐20.30
**Patient residence**	
Metropolitan	50 (50.0)
Regional or interstate	50 (50.0)
**Parental country of birth**	
Australia	86 (86.0)
Other	14 (14.0)
**Tumor types**	
Solid	52 (52.0)
Brain	31 (31.0)
Hematological	17 (17.0)
Treatment line	
First	16 (16.0)
Second	35 (35.0)
Third or greater	49 (49.0)
**Disease state**	
Refractory	51 (51.0)
Relapsed	29 (29.0)
Other	20 (20.0)
**Patient diagnoses**	
Brain tumors (*N* = 52)	
LGG^a^	27 (27.0)
HGG^b^	12 (12.0)
Medulloblastoma/PNET	9 (9.0)
Ependymoma	2 (2.0)
Acoustic neuroma	1 (1.0)
Glioma (unspecified)	1 (1.0)
**Solid tumors (*N* = 31)**	
Plexiform neurofibroma	6 (6.0)
Rhabdomyosarcoma	3 (3.0)
LCH	2 (2.0)
Osteosarcoma	2 (2.0)
Hemangioma	2 (2.0)
Hemangioendothelioma	2 (2.0)
Anaplastic large cell lymphoma	1 (1.0)
Atypical Spitz Naevus	1 (1.0)
Clear cell adenocarcinoma	1 (1.0)
Desmoid tumor	1 (1.0)
Germ cell tumor	1 (1.0)
Hepatoblastoma	1 (1.0)
Kaposiform lymphangiomatosis	1 (1.0)
Liposarcoma	1 (1.0)
Melanoma of soft parts	1 (1.0)
Non‐Hodgkin's lymphoma	1 (1.0)
Non‐Langerhans histiocytosis	1 (1.0)
Renal cell carcinoma	1 (1.0)
Spinal Sarcoma	1 (1.0)
Wilm's tumor (Kidney)	1 (1.0)
**Hematological (*N* = 17)**	
Pre‐B ALL	9 (9.0)
AML	2 (2.0)
ETPLL	2 (2.0)
Myelodysplastic/proliferative disorder	2 (2.0)
Hypereosinophilic syndrome	1 (1.0)
Biphenotypic leukemia	1 (1.0)

Abbreviations: AML, acute myeloid leukemia; ETPLL, early T‐cell precursor lymphoblastic leukemia; HGG, high‐grade glioma; LCH, Langerhans cell histiocytosis; LGG, low‐grade glioma; PNET, primitive neuroectodermal tumor; Pre‐B ALL, precursor B‐cell acute lymphoblastic leukemia.

^a^ LGG encompassed diffuse astrocytoma, disseminated glioneuronal tumor, ganglioglioma, low‐grade glioma, oligoastrocytoma, pilocytic astrocytoma and pleomorphic xanthroastrocytoma.

^b^ HGG encompassed glioblastoma multiforme, anaplastic astrocytoma and diffuse intrinsic pontine glioma.

There were 32 unique diagnoses across the patients included in this study (Table 1). Over half were diagnosed with a brain tumor (*N* = 52, 52%); with solid tumors the next most common diagnostic group (*N* = 31, 29%). Eighty percent of patients were experiencing refractory or relapsed disease, and 84% received a targeted drug at their second line of treatment or greater, indicating a relatively pre‐treated population.

### 3.2 Patterns of treatment allocation

The majority of novel drug regimens were prescribed with curative (68/124, 54.8%) or palliative (30/124, 24.2%) intent; other categories included temporizing treatment, where the novel drugs were used to slow the growth of potentially curable disease; and bridging to bone marrow transplantation. Two treatment regimens did not fit into any of these categories: One where treatment intent was undefined, and another where the novel drug was used to enhance standard therapy.

The primary reason cited explicitly by oncologists in their clinical notes for their choice to use a novel drug was genomic results identified through molecular diagnostics or inferred based on clinical diagnosis in the case of NF1 in neurofibromatosis (*N* = 44, 33.1%) (Table S1). The next most frequent category was “reason not clearly documented” (*N* = 23, 17.3%). For seven patients, evidence from the literature or unpublished clinical trials was the primary justification for the allocation of a novel drug, while for 18 patients, novel treatments were trialed due to conventional treatment failure, but no specific evidence was cited (13.5%) (Table 2). For specific indications, such as radiation necrosis, treatment for a bleeding syrinx, or to avoid resistance to targeted agent monotherapy, there was no explicit documented reference to the evidence for the use of the selected drug in that context. Overall, documentation for the specific rationale behind the choice to use novel drugs varied in detail between oncologists and also between documentation prior to and following the introduction of the EMR.

**TABLE 2:** Rationale for novel drug and oncologist prescribing patterns for the 133 novel drugs prescribed in this study

Novel drug rationale (*N* = 133)	*N* (%)
Genomic result	44 (33.1)
Unclear	23 (17.3)
Conventional treatment failure	18 (13.5)
External opinion	10 (7.5)
Acute deterioration	7 (5.3)
Evidence cited	7 (5.3)
Pharmacological therapy preferred	4 (3.0)
Ineligible for clinical trial	4 (3.0)
Parental choice	3 (2.3)
Prior clinical use	3 (2.3)
Combination to prevent resistance	2 (1.5)
Steroid sparing therapy	2 (1.5)
CAR‐T Cell Anergy	1 (0.8)
Treatment continuation	1 (0.8)
Required treatment intensification	1 (0.8)
Loss of major molecular response post transplant	1 (0.8)
Radiation necrosis	1 (0.8)
Recommended for Bleeding Syrinx	1 (0.8)

Prescribing oncologist (Employment status, Specialization)	*N* (% per oncologist)
Oncologist 1 (Full‐time, brain and solid tumors)	38 (28.6)
Oncologist 2 (Full‐time, brain and solid tumors)	18 (13.5)
Oncologist 3 (Full‐time, brain and solid tumors)	15 (11.3)
Oncologist 4 (Full‐time, brain and solid tumors)	14 (10.5)
Oncologist 5 (Full‐time, hematological malignancies)	9 (6.8)
Oncologist 6 (Part‐time, solid tumors)	6 (4.5)
Oncologist 7 (Full‐time, hematological malignancies)	6 (4.5)
Oncologist 8 – 17^b^	<5^a^ (<3.8)

^a^ Twenty seven drugs prescribed in total.

^b^ Oncologists 8‐17 included five full‐time and five part‐time oncologists; seven who specialize in brain and solid tumors and three who specialize in hematological malignancies.

Frequency of prescription also varied between oncologists, with Oncologists 1‐4 collectively accounting for 85 novel drug prescriptions (63.9%) (Table 2). Oncologist 1 alone prescribed 38 (28.6%) of the novel drugs in the study period. The majority of novel drugs were prescribed following discussion at a multidisciplinary meeting (90.2%). A significantly lower proportion (32.3%) was chosen following consultation with external experts, which ranged from discussions with colleagues at other local hospitals, to national meetings and consultations with international experts. Documentation for these consultations was irregular, particularly prior to the introduction of the EMR. They were only occasionally referenced in clinical notes or letters written by the primary oncologist, so it was unclear if these data accurately represented the prevalence of external opinion in treatment decision‐making processes.

### 3.3 Drug pricing and billing

Drug prices were extracted using the pharmacy dispensing records and the EMR. Where prices were unlisted, a pharmacist was consulted for the available pricing. In five regimens, medications such as powdered trametinib, ofatumumab, alpesilib, and veliparib were not available commercially and no pricing could be obtained. Median novel drug cost was $15 521.09 AUD, with prices ranging from $6.53 AUD for one course of liquid sodium valproate used as a histone deacetylase inhibitor, to $258 339.24 AUD for a three‐year course of dabrafenib capsules.

The majority of drugs were hospital‐funded following approval by the DUC (*N* = 60, 45.1%), or accessed through compassionate means provided by pharmaceutical companies (*N* = 52, 39.1%). Patients and their families self‐funded access on five occasions (3.8%) while the source of funding was unclear for the remainder (*N* = 16, 12.03%). Self‐funded medications were relatively inexpensive compared to the other novel drugs, ranging from $6.53 AUD (sodium valproate) to $4581.23 AUD (everolimus). Documentation for patient billing type varied in location within each file and in level of detail.

### 3.4 Institutional approval

Twenty‐seven applications to DUC for off‐label novel drugs were identified for 19 patients. Seventeen patients were among the 100 included in this study and the remaining two were excluded, as meeting entries could not be reconciled with patient records. Of these, 18 applications were accepted. Two were rejected for insufficient evidence provided in the application, and imminent transition to adult care for a 19‐year‐old patient, respectively. Four applications were withdrawn due to patient disease progression, compassionate access obtained elsewhere, and an unspecified reason (*n* = 2). In three applications excluded from the aforementioned counts, the decision of the DUC was not explicitly documented but was inferred from clinical notes or subsequent meeting minutes: two further approvals, and one additional rejection. In all instances, the precise clinical indication for the novel drug was not documented, and clinical context was limited to descriptors such as “urgent” or “for end‐of‐life care.” It was unclear if DUC possessed additional information in attachments, which were not included in the minutes.

Fourteen out of the 18 accepted applications were approved for a set timeframe (doses, cycles, months, or weeks), ranging from a single dose to up to 12 months. Extensions were requested on six occasions for three patients: one had four extensions approved, another had one extension approved, and one outcome was unknown following escalation to the hospital executive. The criteria used by the committee to discuss the extensions were not explicitly documented beyond commentary that the treatment appeared to be working. While progress was discussed for these patients, outcomes were not documented for patients, who received DUC approval but for whom extensions, were not requested. Overall, it was not possible to draw conclusions regarding the decision‐making process of the DUC.

### 3.5 Treatment outcomes

Ninety‐one of 124 treatment regimens with novel drugs (73.4%) achieved an objective response of complete remission, partial remission, stable disease, or mixed response. Response rates were based on documentation by the respective clinical oncologist and were not reported using specified criteria such as the Response Assessment for Neuro‐Oncology (RANO),^9^ or Response Evaluation Criteria in Solid Tumors (RECIST).^10^ Of these 91 responses, 35 were still ongoing at the time of review in June 2020, 55 had ceased and one was lost to follow‐up (Table 3). For 17 patients, no response was documented, mostly owing to patient death or cessation due to intolerance prior to response evaluation. Seven patients who recorded a response also underwent surgical resection and radiotherapy prior to or during their treatment, making it difficult to attribute their response to the novel drug. Similarly, for five of 11 patients who underwent bone marrow transplantation following the novel drug regimen, it was unclear whether response duration was the result of novel drug use or the transplant itself.

**TABLE 3:** Novel drug regimen clinical outcomes sorted by initial treatment goal

*N* = 124	Curative	Palliative	Temporizing	Bridging to BMT	Other	Overall
**Responders (*N* = 91) (%)**						
**Complete remission**	9 (13.2)	1 (3.3)	0 (0.0)	7 (70.0)	0 (0.0)	17 (13.7)
**Partial remission**	22 (32.4)	4 (13.3)	2 (14.3)	1 (10.0)	0 (0.0)	29 (23.4)
**Stable disease**	19 (27.9)	13 (43.3)	9 (64.3)	0 (0.0)	1 (50.0)	42 (33.9)
**Mixed response**	2 (2.9)	1 (3.3)	0 (0.0)	0 (0.0)	0 (0.0)	3 (2.4)
**Ongoing responses (%)**	22 (32.4)	4 (13.3)	7 (50.0)	2 (20.0)	0 (0.0)	35 (28.2)
**Median duration (months)**	40.3	7.9	19.7	25.13	0	24.0
**Range of duration (months)**	3.68‐93.2	0‐23.72	14.39‐37.06	24.8‐25.46	0	0‐93.2
**Ceased responses (%)**	29 (42.6)	15 (50.0)	4 (28.6)	6 (60.0)	1 (50.0)	55 (44.4)
**Median duration (months)**	9.6	5.03	9.4	3.0	21.9	6.7
**Range of duration (months)**	0.76‐27.53	0.2‐33.97	1.74‐18.17	1.25‐25.56	21.9	0.2‐33.97
**Lost to follow‐up (%)**	1 (1.5)	0 (0.0)	0 (0.0)	0 (0.0)	0 (0.0)	1 (0.8)
**Non‐responders (*N* = 33) (%)**						
**Disease progression**	9 (13.2)	7 (23.3)	0 (0.0)	0 (0.0)	0 (0.0)	16 (12.9)
**No response recorded**	7 (10.3)	4 (13.3)	3 (21.4)	2 (20.0)	1 (50.0)	17 (13.7)
**Total regimens reported**	68	30	14	10	2	124

Abbreviation: BMT, bone marrow transplant.

When 54 regimens for low‐grade malignancies with superior prognoses such as acoustic neuroma, desmoid tumor, hemangioma, hemangioendothelioma, langerhans cell histiocytosis, non‐langerhans histiocytosis, plexiform neurofibroma, low‐grade glioma, and atypical spitz naevus were excluded from analysis, 46 of the remaining 70 regimens (65.7%) for high‐grade malignancies resulted in an objective response (Table 4). More specifically, 15 (21.4%) patients recorded complete remission, 13 (18.6%) partial remission, 15 (21.4%) stable disease, and three (4.3%) mixed responses. By contrast, 45 of the 54 regimens (83.3%) for low‐grade malignancies recorded an objective response: Two achieved complete remission (3.7%), 16 (29.6%) achieved partial remission, and 27 (50.0%) achieved stable disease.

**TABLE 4:** Treatment regimen outcomes by grade of malignancy

	Low grade	High grade
**Total (%)**	54^a^	70
**Responders (%)**	45 (83.3)	46 (65.7)
Complete remission	2 (3.7)	15 (21.4)
Partial remission	16 (29.6)	13 (18.6)
Stable disease	27 (50.0)	15 (21.4)
Mixed response	0 (0.0)	3 (4.3)
**Non‐responders (%)**	9 (16.7)	24 (34.3)
Disease progression	3 (5.6)	13 (18.6)
No response	6 (11.1)	11 (15.7)
**Response status (%)**		
Ongoing	25 (46.3)	10 (14.3)
Ceased	19 (35.2)	36 (51.4)
Lost to follow‐up	1 (1.9)	0 (0.0)
**Response duration (months)**		
Median	20.35	6.5
Range	1.7‐91.7	0–93.2

^a^ Low‐grade malignancy encompassed acoustic neuroma, atypical spitz naevus, desmoid tumor, Langerhans cell Histiocytosis, Non‐Langerhans histiocytosis, hemangioma, hemangioendothelioma, kaposiform lymphangiomatosis, low‐grade glioma, and plexiform neurofibroma.

When categorizing outcomes based on initial treating intent, novel drug treatment regimens bridging to a bone marrow transplant recorded the highest proportion of objective responses (8/10, 80%); followed by temporizing treatment to delay progression prior to more definitive, curative therapy, for example, bevacizumab to reduce tumor size rapidly during therapy to minimize functional risks, that is, visual impairment, spinal cord impingement (11/14, 78.6%); then treatment with curative intent (52/68, 76.5%). Patients allocated regimens with palliative intent recorded the lowest response rate, with just 19 of 30 patients responding to treatment (63.3%). The majority of patients allocated treatment for palliation or for temporizing their illness successfully achieved temporary clearance or control of their illness. Bridging to bone marrow transplantation was particularly successful given that in seven cases, patients achieved complete remission, permitting six to undergo bone marrow transplantation despite refractory or relapsed disease. However, 55 of the 91 objective responses recorded eventually ended in progression of disease. Only 22 (42.3%) of the 52 responders allocated a novel drug with curative intent were still experiencing an ongoing response at the time of this audit. The median ongoing response duration for responses ongoing at the time of the audit was 24.0 months (Range: 0 to 93.2 months) compared to 6.7 months for responses which had ceased (Range: 0.2 to 34.0 months). Overall, 71% of novel drug regimens did not work long term: 33 regimens failed to record an objective response (33/124, 26.6%) while 55 resulted in progression of disease despite an initial objective response (55/124, 44.4%) (Table 3). For low‐grade malignancies, 25 out of 45 responses (55.6%) were still ongoing at time of review compared to only 10 of 46 (21.7%) for high‐grade malignancies (Table 4).

**FIGURE 1:**
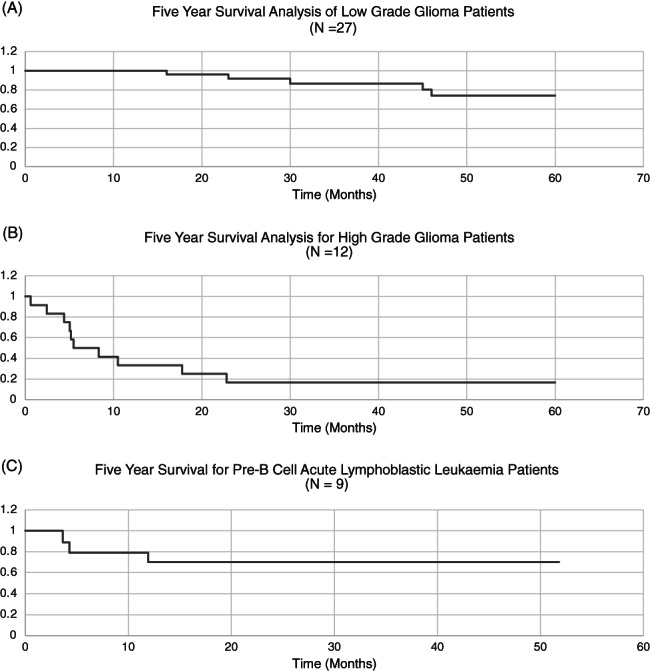
Kaplan Meier Analysis of 5‐year overall survival rates for (A) low‐grade glioma, (B) high‐grade glioma, and (C) precursor B‐cell acute lymphoblastic leukemia.^a^ ^a^No surviving precursor B‐cell acute lymphoblastic leukemia patients could be evaluated for five‐year survival given insufficient data

Five‐year overall survival (OS) rates were also calculated for the three most prevalent diagnoses in this patient cohort: low‐grade glioma (LGG), high‐grade glioma (HGG), and precursor B‐cell acute lymphoblastic leukemia (Pre‐B ALL) (Figure 1). Only 14 LGG, 11 HGG, and three deceased Pre‐B ALL patients had sufficient data for analysis. No surviving Pre‐B ALL patients could be evaluated as none had a sufficient follow‐up duration.

## 4 DISCUSSION

### 4.1 Key findings

Novel drugs were primarily utilized for patients experiencing refractory or relapsed disease (80%) and were prescribed at the second line of treatment or greater (84%, Table 1). The majority were funded by the hospital and compassionate access schemes, with only five regimens funded by patients. These novel drugs were mostly prescribed with the intention to cure (54.8%) or to palliate (24.2%), and almost three quarters of these regimens achieved an objective response. However, more than half of the objective responses (55/91, 60.4%) did not continue past an average of 9 months. The vast majority of novel drug regimens still eventually resulted in progression of disease or no response at all, implying that these novel drugs did not definitively cure these patients. More specifically, while the majority of patients prescribed novel drugs with curative intent experienced objective responses less than half of these responses were still ongoing at the time of the audit (Table 3). Further, since follow‐up durations varied greatly, these latter patients may yet experience progression of disease as more time passes.

A recent study identified patient survival as a primary consideration for patients and their families in approaching treatment decision‐making.^7^ Estimating the benefit of these novel drugs on patient survival was limited in this study by the small sample size, even for the three most prevalent diagnoses of LGG, HGG, and Pre‐B ALL. Five‐year OS following standard‐of‐care treatment has been reported at 90% for LGG,^11^ 15% for HGG,^12^ and 15% to 51% for early and late relapse in Pre‐B ALL, respectively.^13^ Comparing these rates with those observed in this study (Figure 1), novel drugs conferred little to no improvement for LGG and HGG. In fact, five‐year OS was significantly lower for LGG, however, LGG comprises several diagnoses with varying prognoses and children who failed previous lines of treatment were disproportionately represented in this study.^14^ The five‐year OS rate for Pre‐B ALL was promising; however, none of the surviving Pre‐B ALL patients in this study had been evaluated for 5 years. Two Pre‐B ALL patients also received treatment for refractory rather than relapsed disease, potentially affecting comparisons with the published data in relapse. Hence, there was little evidence from this study to substantiate the benefit of novel agents for patient survival in the most prevalent diagnoses within this patient cohort. This information would clearly be significant for families' decision‐making and should be included in the informed consent process.

Conversely, the majority of regimens allocated with the goals of palliation, temporizing treatment or bridging to bone marrow transplantation successfully achieved objective responses (70.4%). These results implied that these agents successfully provided patients with disease control, which may otherwise not have been achieved by conventional treatment, particularly in refractory and relapsed disease. However, it was unclear if objective, but non‐curative, responses correlated satisfactorily with the expectations of patients and their families, as the documentation did not allow this to be assessed. Further studies examining the psychosocial impacts of advanced testing and parental expectations are needed.

### 4.2 Implications for institutional processes

The novel drugs were predominantly funded by the hospital or through drug company compassionate access. However, it was not possible to describe the full basis for hospital decisions to provide funding, due to a lack of clinical context in the DUC minutes and the variability in the detail of oncologists' clinical notes. With regard to the individual decisions to prescribe novel drugs, the primary reasons cited for the use of a novel drug varied between genomic results, conventional treatment failure, and external recommendation. Critically, none of these refers directly to expected benefit to the child. It was also unclear what constituted an appropriate level of evidence for whatever benefit was intended, as the evidence was not described in the DUC minutes and individual oncologists rarely included specific citations in their applications. This raises some ethical considerations, particularly since facilitating access to these drugs with hospital funds, with relatively unsubstantiated benefit, could potentially adversely impact the care provided to other patients who rely on the same public subsidy.^15^

This study shows that the decision‐making process of the hospital for the approval of novel drug use, and the allocation of hospital funding to these novel drugs, was not explicitly documented in the DUC meeting minutes. There is no clear indication that the hospital was aiming to allocate novel drugs in an equitable way, although of course this may have been the case. Additionally, patterns of prescription were observed to vary between oncologists, with four oncologists prescribing over half of the novel drugs included in this study. While this may be because these oncologists primarily treated patients with brain and solid tumors, the most prevalent diagnoses in this study, these observations may further reflect how access to novel drugs could vary based on the individual preference of the treating oncologist. In the absence of guidelines or decision‐making protocols, patient access to novel drugs may have been disproportionately impacted by subjective factors such as the experience or risk‐appetite of the treating oncologist, individual views, and experiences of DUC members, and difference in individual values concerning the importance of maximizing length of life. Since concern about inequitable access to novel drugs is already being expressed,^6^, ^15^ it is important for hospitals to have explicit decision‐making processes, supported by sound documentation.

### 4.3 Study limitations and future directions

As it is retrospective, this study is limited by lack of control for key variables such as follow‐up duration, prior lines of treatment, patient diagnoses, tumor genomic profile, and the precise indications, which prompted the use of novel drugs. Promisingly, large, prospective clinical trials seeking to assign children with novel drugs based on genomic sequencing such as iCat2,^16^ INFORM2,^5^ the PRecISion Medicine for Children With Cancer (PRISM) Trial,^17^ and the Precision Oncology For Young PeopLE (PROFYLE) Trial,^18^ are already underway to further investigate the clinical efficacy of molecularly targeted agents. However, in the absence of prospective, standardized clinical trials with matched controls, which are difficult to achieve given the rarity of these diseases,^3^ introducing more objective protocols for the use of novel agents would help ensure that even their off‐label use can contribute more meaningfully to the current evidence base. Use of protocols with standardized collection and documentation of data would also assist in development of decision‐making frameworks to guide clinicians and DUCs in the use of novel drugs.

This study was also limited in its ability to evaluate whether patient socio‐economic status and other psychosocial factors influence access to novel drugs. More standardized documentation and reporting of psychosocial and demographic data are needed to properly evaluate whether factors such as financial status or linguistic or cultural background may influence family access to these novel drugs and help identify any issues surrounding equity of access in this emerging paradigm.

## 5 CONCLUSION

Novel drugs present a promising solution to improving clinical outcomes for children with rare, relapsed, and refractory malignancies. The results of this study indicate that while using novel drugs off‐label has provided patients with some benefit, more rigorous and standardized approaches to documentation are required to fully characterize the best use of novel drugs, and to advance the ethical and clinical discussions surrounding their complex implications for patients and their families.

## CONFLICT OF INTEREST

The authors have stated explicitly that there are no conflicts of interest in connection with this article.

## AUTHOR CONTRIBUTIONS

All authors had full access to the data in the study and take responsibility for the integrity of the data and the accuracy of the data analysis. *Conceptualization*, L.G., M.M., J.H.; *Methodology*, J.L., L.G., M.M., J.H.; *Investigation*, J.L., S.K.; *Formal Analysis*, J.L., L.G., M.M., J.H.; *Resources*, J.L., L.G., M.M., J.H., S.K.; *Data Curation*, J.L., S.K.; *Writing – Original Draft*, J.L.; *Writing – Review and Editing*, J.L., L.G., M.M., J.H.; *Visualization*, J.L., L.G., M.M., J.H.; *Supervision*, L.G., M.M., J.H.; *Project administration*, L.G., M.M., J.H.; *Funding acquisition*, J.H, M.M.

## ETHICS STATEMENT

The study was granted ethical approval to access patient records through the electronic medical record at the Royal Children's Hospital Melbourne Children's Cancer Centre by the Royal Children's Hospital Melbourne Human Research Ethics Committee (HREC) (HREC number: QA/60416/RCHM‐2019). For retrospective audits, it is not a requirement of the HREC to obtain informed consent from patients or guardians where the audit involves the use of existing de‐identified clinical data with no foreseeable risk to the participants. In this study, the HREC deemed this to be the case.

## Supporting information


**Supplementary Table S1**. Drugs and corresponding genomic results for treatment rationale.

## Data Availability

The data that support the findings of this study are available from the corresponding author upon reasonable request.
