# Supplementary material for: An institutional audit of the use of novel drugs in pediatric oncology
Source: Cancer Rep (Hoboken). 2021 May 3;4(6):e1404. doi: 10.1002/cnr2.1404 (PMC8714541; doi:10.1002/cnr2.1404)
Supplement: Supplementary file 1 — Supplementary Table S1. Drugs and corresponding genomic results for treatment rationale. [file CNR2-4-e1404-s001.pdf]

**Supplementary Table S1: Drugs and corresponding genomic results for treatment rationale.**

| Drug | Genomic Result Cited | N |
|---|---|---|
| Trametinib[a] | *NF1* | 11 |
| Dabrafenib | *BRAF* | 4 |
| Crizotinib | *ALK* | 2 |
| Nivolumab | Recommended for high mutational burden | 2 |
| Sorafenib | *FLT3* | 2 |
| Trametinib | *BRAF-KIAA* Fusion | 2 |
| Alpesilib | *PI3K* | 1 |
| Crizotinib | *ROS1-GOPC* fusion | 1 |
| Dabrafenib and Trametinib | *BRAF V600E* | 1 |
| Dasatinib | *NUP214-ABL1* | 1 |
| Dasatinib | *FGFR1* | 1 |
| Eribulin mesylate | *PI3K* | 1 |
| Everolimus and Ribociclib | *CDKN2a* and *PTEN* loss, *PDGFRA* amplification | 1 |
| Everolimus and Ribociclib | Unspecified finding but recommended post genomic sequencing | 1 |
| Imatinib | *PDGFRA-FLIP1* fusion | 1 |
| Imatinib | *FGFR1* | 1 |
| Ofatumumab | *CD20* | 1 |
| Pazopanib | Unspecified finding but recommended post genomic sequencing | 1 |
| Ruxolitinib | *PAX-JAK2* Rearrangement | 1 |
| Ruxolitinib | *JAK2* | 1 |
| Tofacitinib | *JAK3* | 1 |
| Trametinib | Unidentified but suspected *BRAF* fusion | 1 |
| Veliparib | *BRCA* signature | 1 |
| Venetoclax | *MLL* | 1 |

[a]Some patients were allocated Trametinib for NF1 were allocated on the basis of a diagnosis of neurofibromatosis rather than a genomic testing result.
